# Predicting rice diseases across diverse agro-meteorological conditions using an artificial intelligence approach

**DOI:** 10.7717/peerj-cs.687

**Published:** 2021-09-03

**Authors:** Rutuja Rajendra Patil, Sumit Kumar

**Affiliations:** Symbiosis Institute of Technology, Symbiosis International (Deemed University), Pune, Maharashtra, India

**Keywords:** Agriculture, Artificial intelligence, Plant disease prediction, Agro-meteorological parameters, Artificial neural network, Activation function (AF)

## Abstract

With the aid of a plant disease forecasting model, the emergence of plant diseases in a given region can be predicted ahead of time. This makes it easier to take proactive steps to reduce losses before they occur. The proposed model attempts to find an association between agrometeorological parameters and the occurrence of the four types of rice diseases. Rice is the staple food of people in Maharashtra. The four major diseases that occur on rice crops are focused on this paper (namely Rice Blast, False Smut, Bacterial Blight and Brown Spot) as these diseases spread rapidly and lead to economic loss. This research paper demonstrates the usage of artificial neural network (ANN) to detect, classify and predict the occurrence of rice diseases based on diverse agro-meteorological conditions. The results were carried out on two cases of dataset split that is 70–30% and 80–20%. The various types of activation function (AF) such as sigmoid, tanH, ReLU and softmax are implemented and compared based on various evaluation metrics such as overall Accuracy, Precision, Recall and F1 score. It can be concluded that the softmax AF applied to 70–30% split of dataset gives the highest accuracy of 92.15% in rice disease prediction.

## Introduction

Environmental uncertainty is spanning the world. In the agriculture sector, this becomes one of the major factors causing harm to the crops and in turn leading the farmers towards debts and suicides. Plant diseases are the biggest challenge. Traditionally, diseases in plants were identified by visual examination of the plants by agricultural experts, consultants, or farmers themselves as they have the knowledge to identify the diseases. The other traditional method was laboratory examination where soil parameters such as PH level, moisture, nitrogen are measured. Microscopic examination and serological methods are some other popularly used laboratory techniques. However, these techniques had certain demerits such as time-consuming where diseases were identified when major crop damage was caused, required personnel monitoring, also these techniques were inefficient for huge farms. Hence, there arose a need where farmers could proactively analyze and identify an infection before it comes visible. Therefore, assisting farmers in safeguarding plants from diseases becomes the motivation to automate the plant disease detection processes (Arivazhagan et al., 2013; Kumar & Kaur, 2015; Khirade & Patil, 2015). These automatic techniques discard the demerits of the traditional approach of plant disease detection by making the process faster and more accurate and reducing personnel monitoring.

**Figure 1:**
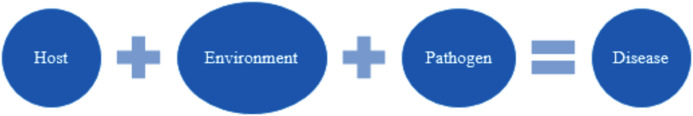
Plant disease causing factors.

Plant disease can occur when all the three factors that are favorable for the disease are present. The factors are the host (the crop to be considered for disease detection), pathogens (factors causing diseases like fungus, virus or bacteria), and environmental conditions (factors that will decide whether the disease will grow or not). If any one of the factors among these is absent, the disease will not occur. Figure 1 represents factors causing diseases in plants.

Rice is an important staple crop of India. India is the second-largest producer of rice in the world and produces 21 percent of the world’s overall rice supply as reported by the United States Department of Agriculture in the year 2018–19. In Maharashtra, rice is the dominant crop of the people grown over an area of 14.99 lakh hectares. The favorable climatic conditions for rice to grow are areas with high temperature, high rainfall, and high humidity, and therefore it is mostly found in southern and eastern parts of India. In Maharashtra, technologies are not reaching most of the farmers. Rice can be harvested throughout the year and that is the reason lots of farmers are farming it. The various diseases like Rice blast, Brown spot, Smut, Bacterial blight are the major occurring diseases on rice. Every year, farmers face huge losses due to these diseases. If the disease is not well predicted or identified in the early stage, then it could lead to huge economic loss to the farmers.

The research paper employs an artificial neural network methodology for the prediction of four different types of diseases in rice along with health conditions for rice growth (Sharma, Singh & Singh, 2018). It is the first foray into the field of rice disease prediction.

## Related Work

In the existing literature for plant disease prediction, it has been observed that prediction of the diseases is commonly made by processing the images, by making use of the Internet of Things (IoT), and prediction based on correlation of agro-meteorological information.

A model to extract prime characteristics and detect the tomato powdery mildew disease on the leaves of tomato plants (Raza et al., 2015) uses thermal and stereo images of the plants. The model (Hernández-Rabadán, Ramos-Quintana & Guerrero Juk, 2014) uses RGB images to detect the same disease. RGB-D sensors are used to detect apple scab Chéné et al. (2012) and Garcia-Ruiz et al. (2013) аirсrаft-based sensors are used to detect the same disease. Mandal et al. (2015) A model for predicting the presence of yellow leaf curl virus in tomato plants. This model employs a support vector machine (SVM) pipeline classifier for classification. The proposed system has focused on identifying eight different types of diseases on cotton leaves. They have named their proposed model as Homogeneous Pixel Counting Technique for Cotton Diseases Detection (HPCCDD) Algorithm. The images are captured in real-time using sensors on the field. Segmentation methods such as Sobel edge detection and Canny edge detection are used to distinguish the disease affected areas of the leaf. The type of the disease and its recommendations are provided as output to the farmers (Revathi & Hemalatha, 2014). A model has been proposed to detect diseases on apple leaves. The captured RGB image is converted to HSI, YUV and grayscale models. Noise is removed based on the threshold value. A region growing algorithm (RGA) is used for the segmentation of the image. To select the most important features from the total of 38 features to increase the accuracy, the genetic algorithm (GA) is combined with correlation-based feature selection (CFS). The SVM classifier is used for classification. All experimental results were performed on MATLAB 7.6. The model produces 90% classification accuracy (Chuanlei et al., 2017). A model is designed based on image processing feature extractors such as ORB, SIFT, HoG, SURF for the detection of corn diseases. These features are combined with SVM, NB, RF, DT classifiers. After evaluation, it was observed that these extractors do not perform well as compared to RGB. RGB when combined with SVM classifiers produces better accuracy in the classification of diseases. The future scope defined by the authors was using features in a combined approach (Kusumo et al., 2019). An image processing and IoT-based techniques and prediction models for locating, detecting, and categorizing diseases on tomato plants are surveyed (Verma, Chug & Singh, 2018). The Android-based application has two objectives, the first being detecting cotton plant leaf diseases and the second being monitoring the quality of the soil. SVM classifier is used to identify the diseases in cotton plants. For soil quality monitoring four sensors for soil moisture, temperature, humidity, and water sensors are interfaced with the Raspberry Pi kit. A relay is used to control external devices such as the sprinkler assembly which is used to sprinkle fertilizer. Water sensors are used to measure the water level in the tank. Overall accuracy for the proposed system is 83.26% (Sarangdhar & Pawar, 2017). A system based on IoT is capable of sending real time data related to the environment such as air temperature, Relative Humidity, moisture, wind speed, rainfall, sun intensity to cloud storage. SVM machine learning classifier is used to predict the spread of fungal diseases on the local crop (Truong, Dinh & Wahid, 2017). A novel approach for preventing groundnut crop disease is proposed, which is based on IoT and machine learning (ML). Humidity and temperature sensors are used to check the humidity and atmospheric temperature of the plant, while soil moisture sensors are used to determine the status of the soil. Sensors, webcams, GSM, and controllers are all used to receive data from the groundnut farm. Using the XG boost machine learning model, the received data is analyzed and predicted for every possible disease occurrence. Farmers are notified of the рrеdiсtiоn through the internet (Yoganand et al., 2020). The model called the SISALERT forecasting scheme is developed (Fernandes, Willingthon & Sanhueza, 2011). It is a web-based model in general. The program uses risk management models to track the weather hour by hour and collect station data. The weather data were analyzed using previous/recent dataset reports and the probability of disease prediction. The coupling of crop and disease models is used to run this model. This is a one-of-a-kind feature of the SISALERT scheme.

Nowadays, data mining techniques are used in almost every area, including medicine, agriculture, the atmosphere, and technology. A clustering approach for monitoring weather-based meteorological data is developed (Shobha & Asha, 2017). A forecasting model based on fuzzy logic structures to estimate the severity of crop diseases. The linguistic variables are described by fuzzy logic. These variables make it easier to forecast reliable, expected outcomes (Sibiya & Sumbwanyambe, 2019). An ANN-based approach for predicting the type of crop that can be cultivated in a particular region based on soil and weather parameters. PH level, soil type, sodium, hydrogen, potassium, biomass, and other micro and macronutrient parameters are used. The input values to the model are values of environmental attributes such as rainfall, humidity, temperature. The algorithms employed are Back propagation and feed forward algorithms. For error estimation, the back propagation process employs gradient descent. Later, this algorithm is applied on sugarcane, cotton, jowar, corn, rice, wheat, groundnut, and soyabean crops. As a simulation technique, MATLAB is used (Anitha & Chakravarthy, 2018). An analysis was conducted in which an ANN method was used to estimate crop yield. The crop considered for the study was an onion. The neural network has a 1–2–1 configuration, with one input layer neuron, two hidden layer neurons, and one output layer neuron. Back propagation approach is used. Non-linear regression is used to forecast the outcome. The results show that ANN outperforms the regression approach in terms of accuracy in predicting crop yield. Three various ANN versions are also used. The model with structure 1–3–1 is more reliable than the regression model, according to the results. Changing the number of hidden layer neurons in the model has little effect on the system’s accuracy (Šťastný, Konečný & Trenz, 2011). The model named BLITE-SVR was developed for the potato late blight prediction model, with the help of which they predicted and validated the primary date of prevalence using data from 1976 to 1985 and 2009 to 2012 using support vector regression (SVR), a statistical approach that provides adequate results. They gathered 13 climatic variables for the forecast and found that they had a strong association with the first date of late blight occurrence. The authors assessed BLITE- SVR’s results using the conventional shifting-common methodology, as well as speed regression and linear regression. The accuracy of estimation for the first date of occurrence increased to 64.3 percent using BLITE-SVR, indicating a higher level of accuracy than 42.9 percent using the conventional moving-common method, 42.9% using tempo regression, and 35.7% using linear regression (Gu et al., 2016).

The authors (Alagumariappan et al., 2020) have developed a real time decision assistance system using a Raspberry PI microcontroller. The model considered three different types of plant leaves namely *Nerium oleander*, *Ixora coccinea*, and sweet scented geranium for disease identification. The comparison analysis of three machine learning classifiers such as extreme learning machine (ELM), SVM with linear, and SVM with polynomial kernels was analyzed. When compared to the SVM classifiers, the results show that the ELM performs better as 95% accuracy is achieved. The researchers (Iniyan et al., 2020) have studied two widely used machine learning algorithms namely SVM and ANN for plant disease identification. The common diseases that occur in Indian crops such as blight, cankers, rust, and wilt are focused. SVM proves better when time and space complexity is considered however when accuracy is considered ANN models work better.

It can be derived from the aforementioned literature review that the majority of the work done is in foreign countries suitable for their geographical climatic conditions. As per the survey done (Kaur, Pandey & Goel, 2019), plant disease prediction is still in its infant stage in India. Images were primarily used for disease classification in plants. The diseases of fruits and vegetables were more explored (Fenu & Malloci, 2021). ANN is included in this article, which is one of the Artificial Intelligence methods available. The literature indicates that ANN-based models have been successfully implemented and perform well in other agricultural domains. Also, ANN has never been used to predict different types of rice diseases based on climatic parameters.

## Materials & Methods

This section comprises data set collection, pre-processing of the dataset, and methodology implemented for the proposed work in the paper.

### Dataset collection

The dataset was collected for Dapoli taluka of Ratnagiri district (Maharashtra, India) which is one of the high productivity districts for rice. The dataset is compiled from the data obtained from the online visual crossing weather platform, the Indian Meteorological Department, and All India Coordinated Research Project on Agrometeorology (AICRPAM). The dataset is composed of 1,634 rows of weekly weather data from the year 1989 to 2019 as per Indian Meteorological Department (IMD) with eight numerical input weather parameters and one numerical target variable. The parameters such as Temperature, Relative Humidity, Precipitation, Wind speed, etc. are comprised in the dataset. The dataset involves predicting the occurrence of five different classes of rice diseases based on the weather parameters in the district. To predict a particular value of a particular parameter the algorithm needs to learn from available historical data. The algorithm understands the correlation among different parameters in the dataset.

### Dataset pre-processing

A data visualization Python library called Seaborn is used to find significant characteristics in the dataset. It uses the SNS.heatmap( ) function to build a heat map as an output. It shows a correlation matrix for different combinations of agro-meteorological variables in the dataset. The range of correlation values is 0 to 1, with the values above 0.3 and below −0.3 indicates a significant relationship. The data was pre-processed after the features were selected. There were alpha-numeric values in certain fields, such as the date field in the dataset so before processing, these data must be transformed to a single numeric value. The label encoder( ) function from the Pandas library was used for this. The dataset was having null values so to remove these null values, the function isnull( ) was invoked. The data are pre-processed through standardization, which changed the actual scale of the input values to an interval between 0 and 1.

### Model adopted based on ANN using Keras for predicting weather parameter values

Initially, a sequential model by adding a sequence of layers is created. A fully-connected network structure with three layers is used. They are defined using the dense class. The number of neurons in the layer is the first argument and the activation function using the activation argument is the second argument. The next step is to select an activation function from sigmoid, ReLU, softmax and tanH. As the number of inputs is 8 parameters, the input layer and the output layer will have eight neurons for predicting weather parameter values. After creating the model, it is necessary to compile a model. In this step, it is necessary to specify the loss function to evaluate a set of weights and the optimizer to search through different weights for the network. Here, mean squared error is used as loss function and optimizer used is adam. The metrics used is MAE for prediction of values and accuracy, precision, F1 score for classification of rice diseases. The model is trained or fit on the loaded data by calling the fit( ) function on the model. Training occurs over epochs and each epoch is split into batches. The number of epochs considered here is 500 and batch size is 32. Lastly evaluation of the model is done. The evaluate**( )** function is used to perform the same. It will return a list with two values. The first will be the loss of the model on the dataset and the second will be the accuracy of the model on the dataset.

### Dataset splitting

The historical weather data obtained were divided into two sets, namely the training set and training set. Initially, the artificial intelligence (AI) model is built on a dataset called a training set and the built model is tested on a new set called the test set. The newly developed ML model is applied to test the dataset to measure the performance. This dataset was further split in two different ways. As per the bifurcation of the dataset, if 70–30% split is considered then the data values for the year field that are less than equal to 2010 and greater than equal to 1989 will be considered for training. The data values for the year field that are greater than 2010 and equal to 2019 will be considered for testing. If 80–20% split is considered, then the data values for the year field that are less than equal to 2013 and greater than equal to 1989 will be considered for training and the data values for the year field that are greater than 2013 and equal to 2019 will be considered for testing. Table 1, shows these two variations in dataset division.

**Table 1:** Two cases of data split considered in the manuscript for implementation, summary of activation functions, MAE values for two cases of data split for four activation functions, comparison based on overall accuracy of the model for different activation functions.

% of train–test dataset split	Total weather instances	Training instances	Testing instances
70–30%	1,643	1,150	493
80–20%	1,643	1,313	329

### Algorithm of proposed work

The proposed model is a combination of regression and classification. ANN model is used for implementation. Following are the algorithmic steps of the model.

Step 1: Collect week-wise data of agro-meteorological parameters for disease forecasting.

Step 2: Load data by using the pandas library

Step 3: Perform data pre-processing on the instances of the dataset.

Step 4: Build a model based on ANN using Keras.

Step 5: Divide the dataset into two parts: training dataset and test dataset. The dataset is split into two cases (70–30%, 80–30%) and results are verified accordingly.

Step 6: Training the network by utilizing the dataset.

Step 7: Prediction of future values of climatic parameters.

Step 8: Evaluation of the prediction model

Step 9: Prediction of crop disease occurrence. The final output will be in the form of five classes namely Healthy, Rice Blast, Blight, Brown Spot, and False Smut.

Step 10: Evaluation of the classification model.

Class 1—Healthy, Class 2—Rice Blast, Class 3—Bacterial Blight, Class 4—Brown Spot, Class 5—False Smut.

### Artificial neural network

The biological nervous system influenced the creation of the ANN. It is made up of closely connected nodes known as neurons. It is a network made up of artificial neuron clusters that are linked together with different weights allocated to each connection. There are certain learning laws that determine the states of the neurons as well as the values of each relation. There are two types of learning namely supervised and unsupervised learning. Supervised learning is utilized in the proposed approach. The relationship between input and output variables is determined using ANN, a non-linear mathematical procedure. A neuron’s computational model can be defined as:

Initially, the n inputs are denoted as

(1) $${\rm X} = {x_{\rm{1}}}, {x_{\rm{2}}},...... {x_{\rm{n-1}}},\; {x_{\rm{n}}}$$

These inputs are fed into a neural node, where they are multiplied by their weights, which are denoted by

(2) $${\rm W}=w_1,\;w_2, ..... {w_{{\rm{n}} - 1}},\; {w_{\rm{n}}}$$

The results of these multiplications are then added together. The summed value is denoted by Sum variable.

(3) $$Sum = X\;{\rm{\times}}\;W$$

(4) $$Sum = \sum\limits_{i = 1}^n {{x_i}} \;{\rm{\times}}\;{w_{\rm{i}}}$$

After that, the sum value is transferred into an activation function denoted by φ. So output can be defined as:

(5) $$Y(output) = {\rm \phiv} ({\rm{Sum}})$$

**Figure 2:**
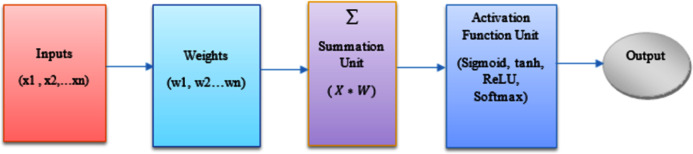
Overview of an ANN model.

Along with the actual inputs to the node, an external bias also known as the threshold term denoted by θ, is applied to the node, which has the effect of decreasing or increasing the net input to the node, based on its sign. The bias can be modeled as a constant zeroth input ${x_0}$ = 1, and an associated weight ${w_0}$ = θ whose value can be negative, positive, or zero. Figure 2, represents the overview of the ANN model used in this paper.

The Back propagation algorithm is used in the proposed process, which operates on the concept of error correction learning law. The basic one-layer perceptron can only solve linearly separable problems, but it has trouble with linearly dependent scenarios. The partial derivative of the error is taken considering the corresponding weight of the network. This helps to understand how the error on the network is going. if we add the weight to the negative of this error, the error will minimize and shift towards the local minima. This algorithm is known as back propagation since it begins from the output layer, moves to the hidden layer, and then back to the input layer in a backward direction. There have been several weather-based models produced for different types of crops and different diseases. Potatoes, tomatoes, and grapes are the major crops for which the forecast model has been proposed in the literature. They are focused on multiple linear regression analysis. However, the ANN method has never been used to forecast multiple diseases in rice.

### Activation function

It is an internal state of a neuron. It is used to convert the input signal on a node of ANN to an output signal. It is a weighted sum of input that becomes an input signal to the activation function to give one output signal. They introduce non-linear properties to the network. Therefore, it helps to solve complex problems that include images, videos, and audio. The accuracy of the model is majorly dependent on the activation function. Many types of activation functions are available. However, in the proposed model, four different AF are demonstrated namely sigmoid, hyperbolic Tangent (TanH), ReLU, and softmax. In the regression part, the activation function is applied to hidden layers as the model predicts the value of climatic variables. For classifying the rice diseases these classifiers are evaluated by applying at output layers.

#### Sigmoid

The sigmoid function can be represented as

(6) $${\bf{sigmoid}}\left( {\bf{x}} \right) = {{\bf{1}} \over {{\bf{1}} + {{\bf{e}}^{ - {\bf{x}}}}}}$$

**Figure 3:**
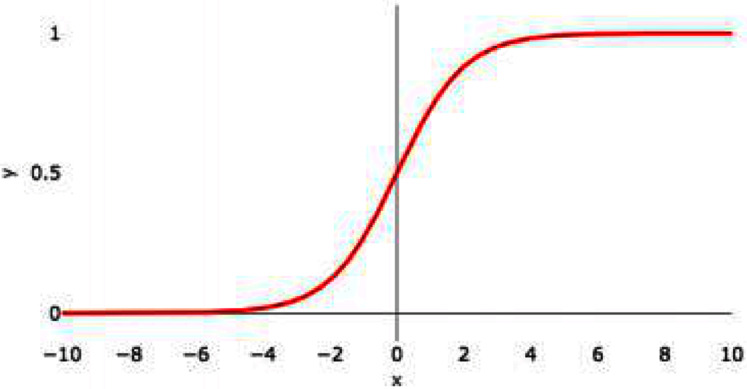
Sigmoid activation function graph.

Figure 3, shows the graphical representation of the sigmoid activation function.

This function converts the input into a range from 0 to 1. The sigmoidal graph is having S-shaped curve. The product is any value, by applying a sigmoid function, the value comes in the range of 0 to 1. If the result is less than 0.5 then consider the value as 0 and if the result is more than 0.5 then consider the value as 1. If the value obtained is 0.5 then mark it as 0. The center of the curve lies at 0.5. The value 1 means the neuron is activated and is transferring the signal and is helping in classification.

(7) $${\bf{f}}\left( {\bf{x}} \right) = \left\{ {\matrix{ {0\;\;\;\;\;\;\;{\bf{if\;x}} \lt 0} \cr {1\;\;\;\;\;\;\;{\bf{if\;x}} \gt 0} \cr {0.5\;\;\;\;{\bf{if\;x}} = 0} \cr } } \right\}$$

This function eliminates outliers or extreme values in data without replacing them. It is used in models where there is a need to predict the probability of an output. It is best suited for multiclass classification.

### Hyperbolic tangent (tanH)

**Figure 4:**
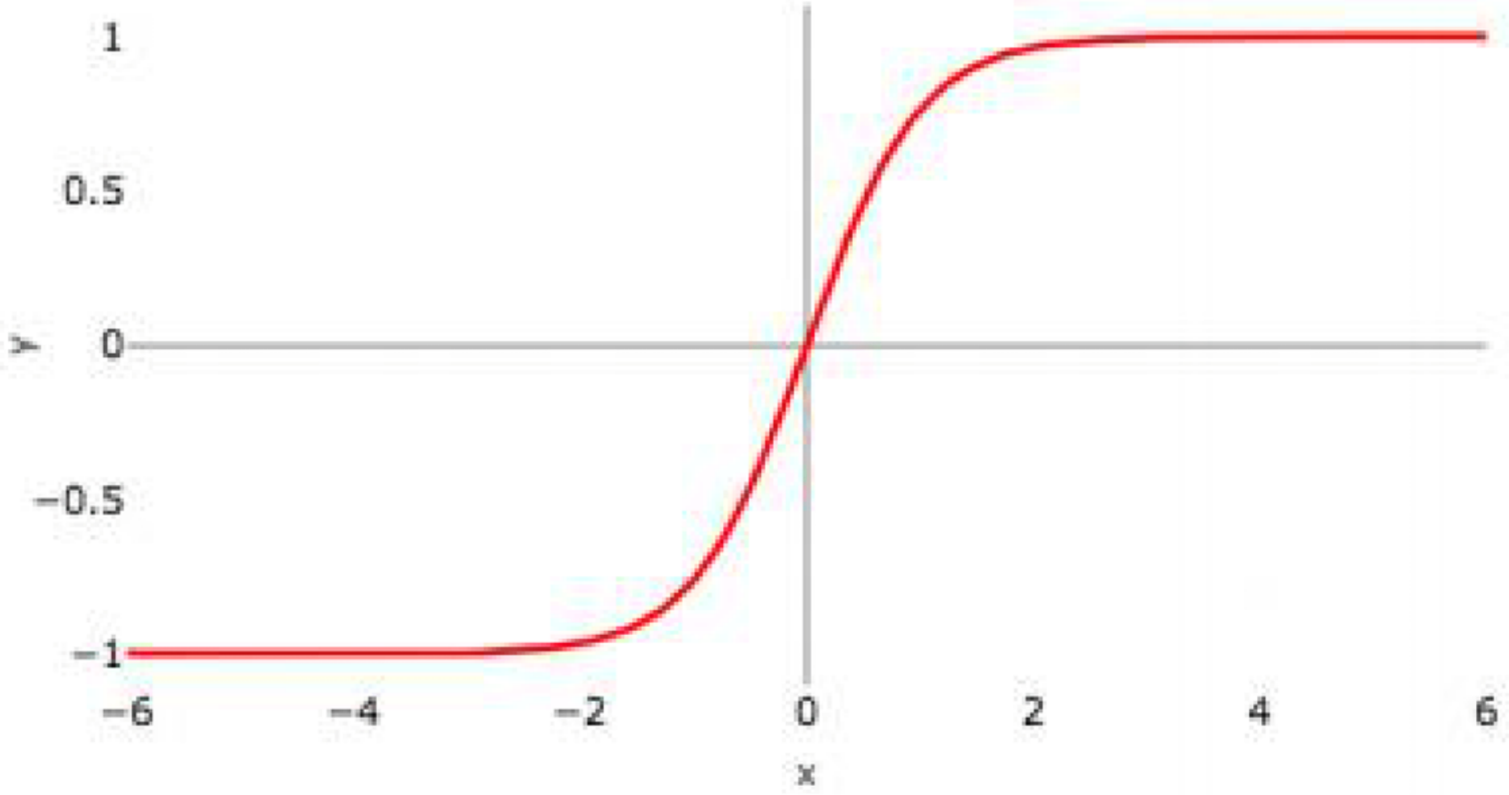
tanH activation function graph.

Hyperbolic tangent is commonly termed as tanH function. It is similar to Sigmoid AF; however, tanH AF has better performance as the tanH AF ranges between −1 to 1. It is a nonlinear AF. It is centered at 0 so it is symmetric around the origin. Figure 4 represents the graph of tanH AF. It also has S-shaped curve similar to sigmoid AF.

tanH can be represented as follows:

(8) $$\tanh \left( {\bf{x}} \right) = {{{{\bf{e}}^{\bf{x}}} - {{\bf{e}}^{ - {\bf{x}}}}} \over {{{\bf{e}}^{\bf{x}}} + {{\bf{e}}^{ - {\bf{x}}}}}}$$

It also faces a vanishing gradient issue similar to sigmoid AF. tanH activation function can be represented by the following mathematical model

(9) $${\bf{f}}\left( {\bf{x}} \right) = \left\{ {\matrix{ {-1\;\;\;\;\;\;\;\;\;{\bf{if\;x}} \lt 0} \cr {1\;\;\;\;\;\;\;{\bf{if\;x}} \gt 0} \cr {0\;\;\;\;\;\;{\bf{if\;x}} = 0} \cr } } \right\}$$

If the input is a negative value then it will map to -1, if the input values are positive then the output will be mapped to 1 and if the input is 0 then it will be mapped to zero itself.

### ReLU

**Figure 5:**
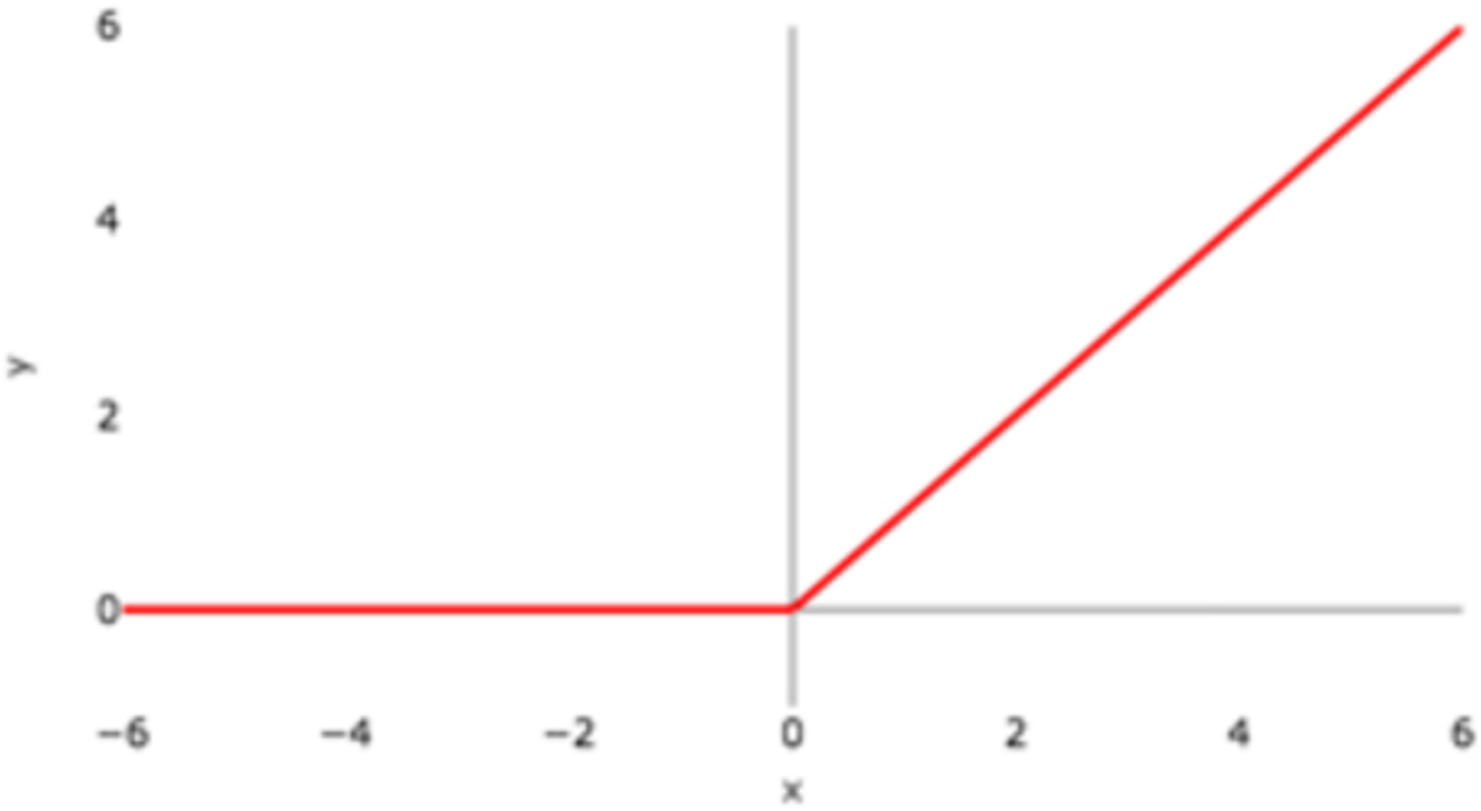
ReLU activation function graph.

ReLU stands for Rectified Linear Unit. Figure 5 represents the graphical representation of the ReLU activation function. The graph has a linear nature.

ReLU activation function can be represented by the following mathematical model

(10) $${\bf{f}}\left( {\bf{x}} \right) = \left\{ {\matrix{ {\bf x} & {{\bf {if\ x}} \ge 0} \cr {{0}}\ & {{\bf{if\ x}} \lt 0} \cr } } \right\}$$

The above ReLU mathematical model says that if the input value is a negative value then the output value will be 0 and if the input value is a positive value then the output will be the inputted positive value itself. As a result, it essentially eliminates the negative aspect of the feature. It can also be represented as

(11) $$\rm ReLU(y) = max(y,0)$$

where y is the product of the weight of the neuron and the input value.

If the value of y is negative, then the output is transformed to 0 and if the value of y is positive, then the output is a positive number itself. If the value of y is negative, then the maximum of a negative number and zero is zero and if the value of y is positive then the maximum of a positive number and 0 is a positive number itself. ReLU removes the vanishing gradient limitation experienced by tanH and sigmoid activation functions.

### Softmax activation function

The output produced with this activation function ranges between 0 to 1. It generates a multiclass probability distribution as a vector over target classes and the summation of the probabilities equals 1. Softmax activation function can be represented as

(12) $${\bf{Softmax}}\left( {{{\bf{x}}_{\bf{i}}}} \right) = {{\exp \left( {{{\bf{x}}_{\bf{i}}}} \right)} \over {\sum\limits_{\bf{j}} {\exp } \left( {{{\bf{x}}_{\bf{j}}}} \right)}}$$

The higher the value in the vector means higher is the probability of the output mapping to a particular class. It is typically used in multiclass classification and performs best if used in the output layer of the network. Table 2 summarizes the activation functions used in the research paper.

**Table 2:** Summary of activation functions.

Activation function	Equation	Graph
Sigmoid	$f\left( x \right) = \left\{ {\matrix{ {{0}} {if\ x \lt 0} \cr {1} {if\ x \gt 0} \cr {0.5} {i\ fx = 0} \cr } } \right\}$	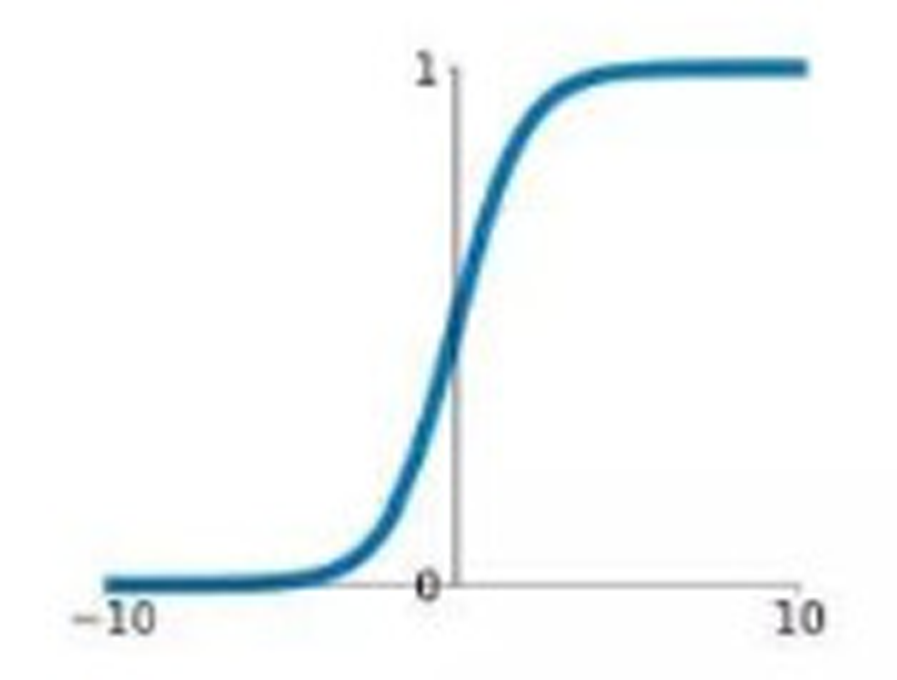
ReLU	$f\left( x \right) = \left\{ {\matrix{ {x} {if\ x \ge 0} \cr {0} {if\ x \lt 0} \cr } } \right\}$	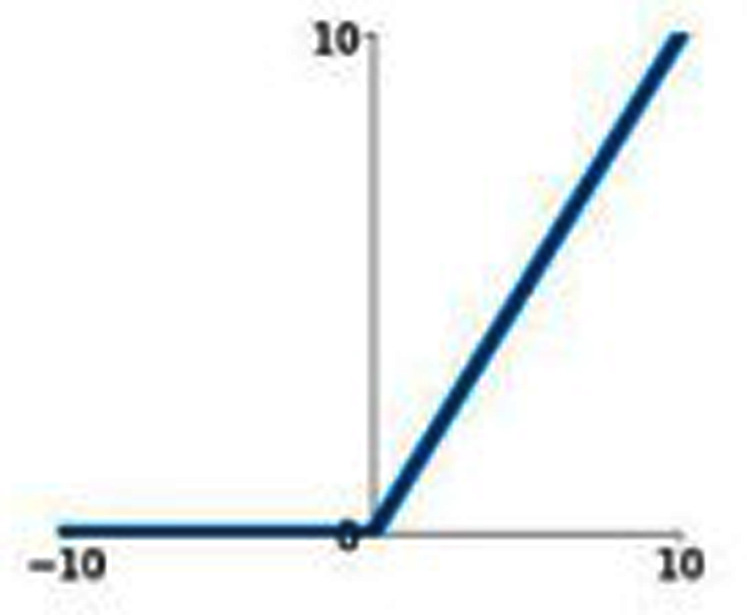
Softmax	${\bf{Softmax}}\left( {{{\bf{x}}_{\bf{i}}}} \right) = {{\exp \left( {{{\bf{x}}_{\bf{i}}}} \right)} \over {\sum\limits_{\bf{j}} {\exp } \left( {{{\bf{x}}_{\bf{j}}}} \right)}}$	This is not a function of a single fold from the previous layer
tanH	$f\left( x \right) = \left\{ {\matrix{ { - 1} {if\ x \lt 0} \cr {1} {if\ x \gt 0} \cr {0} {if\ x = 0} \cr } } \right\}$	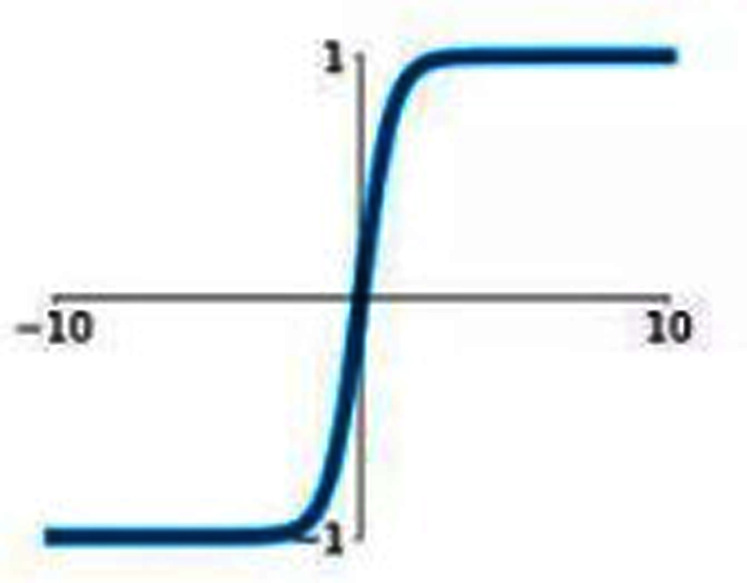

## Results

### Evaluation metrics for the regression model

In the proposed framework, the input to the model is the agro-meteorological parameters collated as per the Indian Meteorological Department week wise for past 30 years. Based on these values, the new values for the parameters are predicted by the model for the future date provided. Once the new values are predicted by the model for the said period, these values are further considered for predicting the rice diseases. This is where regression is applied. There are various performance metrics used to measure the accuracy of the regression model such as Mean Absolute Error(MAE), Mean Square Error(MSE), Root Mean Square Error(RMSE), etc. (Kaundal, Kapoor & Raghava, 2006). An error can be defined as the difference between the actual value and the predicted value by the built model (Piekutowska et al., 2021). The evaluation parameter used to measure the regression accuracy for the proposed model is the mean absolute error (MAE).

(13) $${\bf{MAE}} = \sum\limits_{{\bf{i}} = {\bf{1}}}^{\bf{n}} {{{|{\bf{predicted\ value}}({\bf{i}}) - {\bf{actual\ value}}({\bf{i}})|} \over {\bf{n}}}}$$

where n is the number of instances considered in the climatic dataset.

The mean absolute error is the mean of the sum of all the absolute values of the error. It is simply calculated by averaging the errors. As there are outliers in the climatic dataset that we have used in the research model, MAE is the best suitable metric to measure the regression accuracy of the model. An outlier is a data point in a dataset that lies outside the overall distribution of the dataset. As there is variability in the collected climatic dataset, outliers are present in the dataset. After fitting the model, the performance of the model is assessed by comparing the model predicted values of the agro-meteorological parameters to actual data in the climatic dataset. The lower the value of MAE higher is the crop disease prediction accuracy. If MAE has a value equal to zero, then model predictions are perfect.

### Hyperparameter of ANN

**Figure 6:**
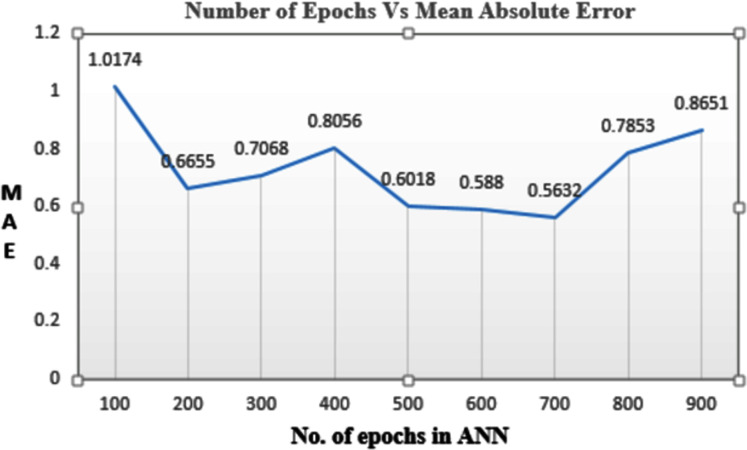
Mean absolute error corresponding to the number of epochs.

Epochs are the number of times the data is given to the neural network. Here the performance of the model was observed on various combinations of epochs to get minimum loss in the predicted values. It was observed that if the epoch was set to 100 the data was under fitted. If the number of epochs was set to 500 then the MAE value is 0.6018. If epochs are set to 700, a minimum MAE value of 0.5632 is obtained, but the time required to train the model was more. So 500 epochs are selected as an optimal solution. Figure 6 shows values of MAE for the various number of epochs. Batch size is the segmentation of the whole data into smaller parts so that the data can be processed individually. Here the batch size is 32. Optimizers are used to minimize the loss so that the model performs better. Adam is the optimizer used to build the model.

Conventionally, an artificial neural network comprises three different types of layers namely input layer, hidden layer, and output layer. The number of input layers used to build the model is eight as the number of features in the dataset is eight, the number of hidden layers used is two. The number of neurons used in hidden layers was selected in such a way that too large neurons would overfit the data and too low number of neurons would underfit the data. There are three thumb rules to calculate the number of neurons in hidden rules first being the number of hidden neurons should be between the size of the input and output layer. The second rule is hidden neurons should be 2/3 the size of input plus output layer size and the third is the number of hidden neurons that should be less than twice the size of the input layer. The third rule is used in the proposed model to calculate the number of hidden layers. Therefore, the number of hidden layers used is 15. The output layer must be equal to the number of target classes. In the proposed model, the number of target classes is five so there are five neurons in output layers. So, precisely (8–15–5) is the topology of the model (Abdipour et al., 2019). After the optimized number of epochs have been obtained MAE is calculated for four types of AF and for two cases of dataset split. Table 3 shows the MAE values for different AF with different data split.

**Table 3:** MAE values for two cases of data split for four activation functions.

Activation function	Number of epochs	MAE 70–30%	MAE 80–20%
Sigmoid	500	0.7265	0.7306
ReLU	500	0.4610	0.5072
Softmax	500	0.7041	0.6724
tanh	500	0.7563	0.7074

It can be observed from Table 3, that ReLU AF outperforms other AF to predict agro-meteorological parameter values if used with a 70–30% dataset split.

### Evaluation metrics for classification model

In our model, true negatives (TN) are the instances where the plants did not have the disease and the model also predicted that plants are not affected by the disease. True positives (TP) are cases in which the plants have diseases and our model estimated that they will have it as well. However, there are several circumstances in which the plants are not affected by the diseases but our model predicts that they are affected. This is known as false positives (FP) and also known as a Type I Error. Similarly, in some circumstances, the plants do have a disease, but our model predicts that it is not affected by the disease. This is known as false negatives (FN) and is also called a Type II Error.

Precision: It is the percentage of plants that are accurately classified as having disease out of all those who have it based on weather parameters. In terms of mathematics:

(14) $${\bi{Precision}} = {{{\bi{TP}}} \over {{\bi{TP}} + {\bi{FP}}}}$$

Recall: The recall is a test of how well our model detects true positives. As a result, recall shows us how many instances were accurately reported as having plant disease out of all those that have it. In terms of mathematics:

(15) $${\bi{Recall}} = {{{\bi{TP}}} \over {{\bi{TP}} + {\bi{FN}}}}$$

F1 score: In our model, the instances that were wrongly diagnosed with plant disease are equally critical because they may be signs of another plant disease, therefore achieving not only a high recall but also a high precision is important. In such instances, we use a technique known as F1-score.

(16) $${\bi{F1}}{\mkern 1mu} {\bi{Score}} = {{{\bi{Precision}}\ *\ {\bi{Recall}}} \over {{\bi{Precision}} + {\bi{Recall}}}}$$

The more nearer the F1 score is equal to 1 the more accurate is the model.

Accuracy: The ratio of the total number of accurate predictions to the total number of predictions is known as accuracy.

(17) $${\bi{Accuracy}} = {{{\bi{TP}} + {\bi{TN}}} \over {{\bi{TP}} + {\bi{FP}} + {\bi{TN}} + {\bi{FN}}}}$$

### Comparison of Activation functions with 70–30% dataset split

**Figure 7:**
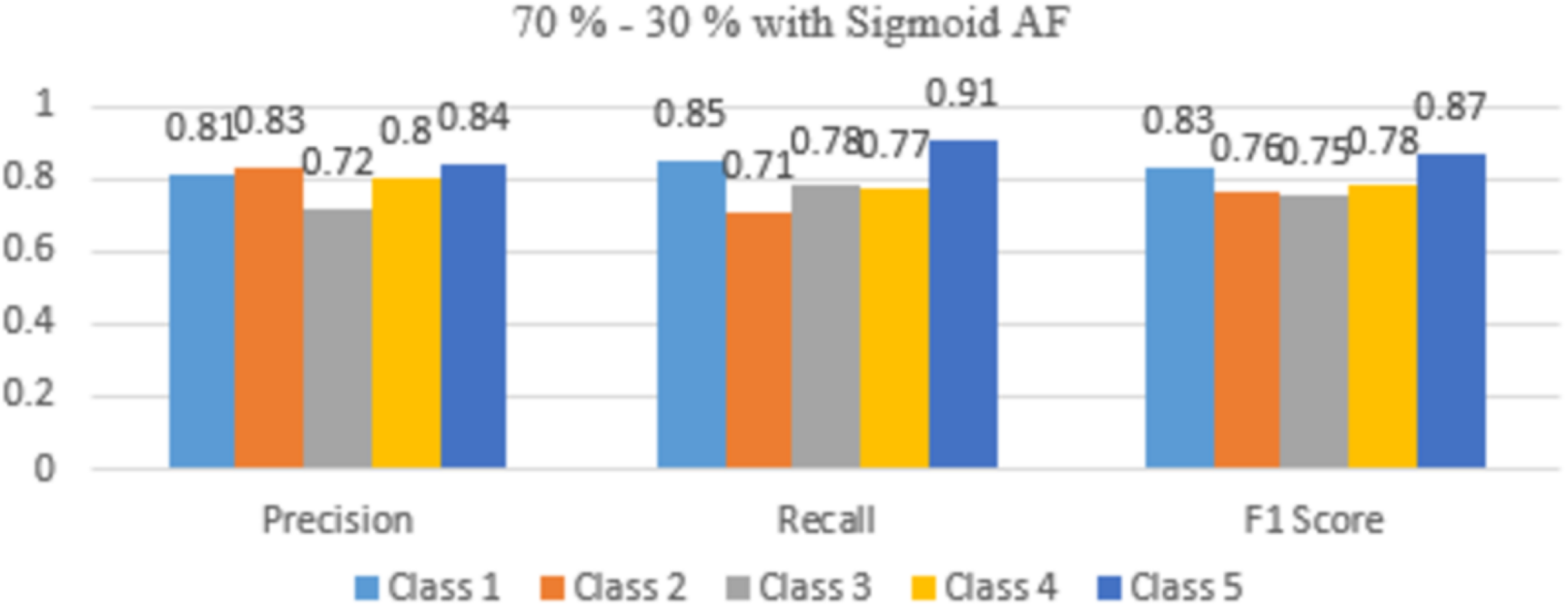
Classification evaluation metrics for 70–30% data split with sigmoid activation function.

**Figure 8:**
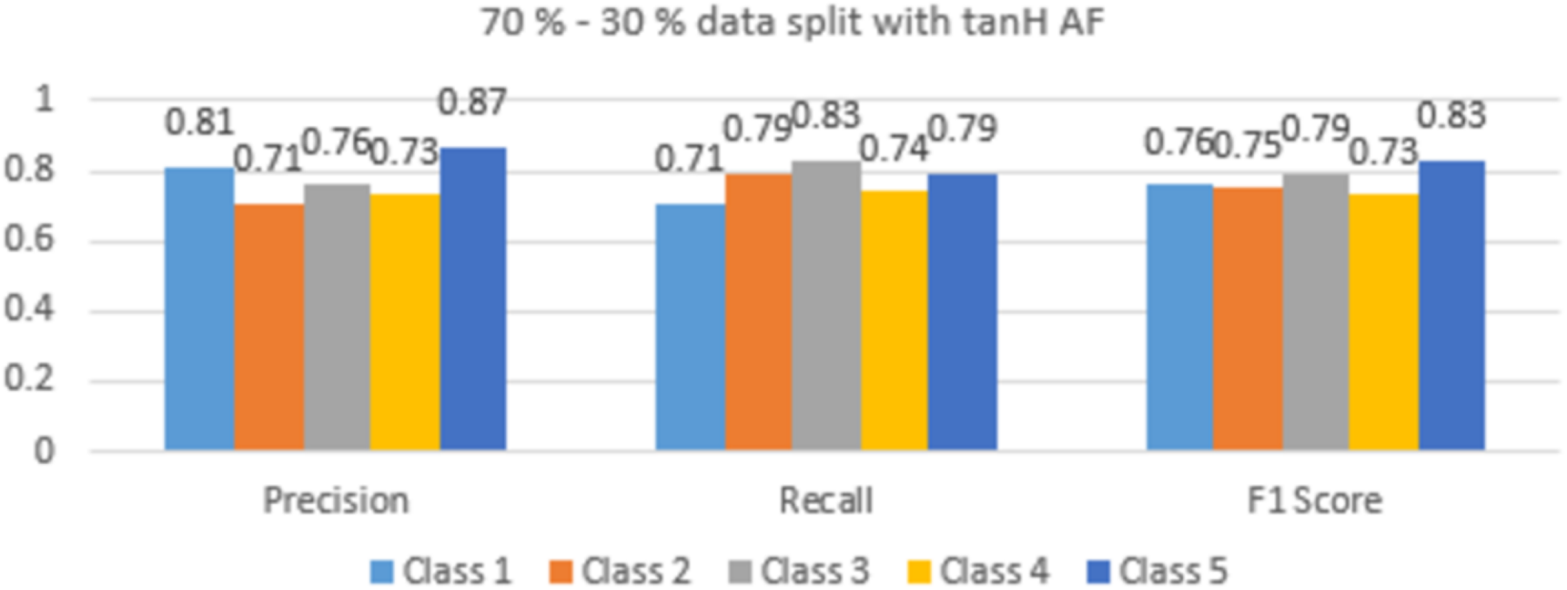
Classification evaluation metrics for 70–30% data split with the tanH activation function.

**Figure 9:**
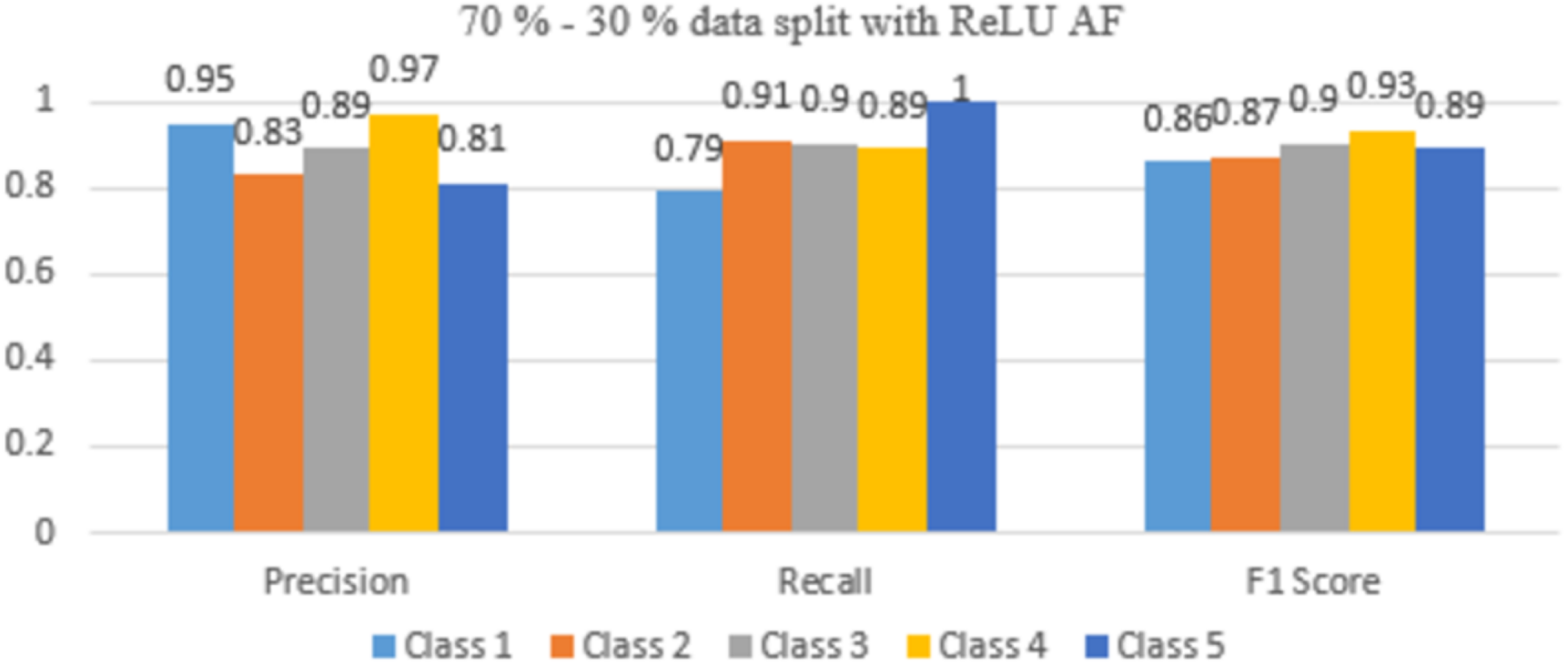
Classification evaluation metrics for 70–30% data split with the ReLU activation function.

**Figure 10:**
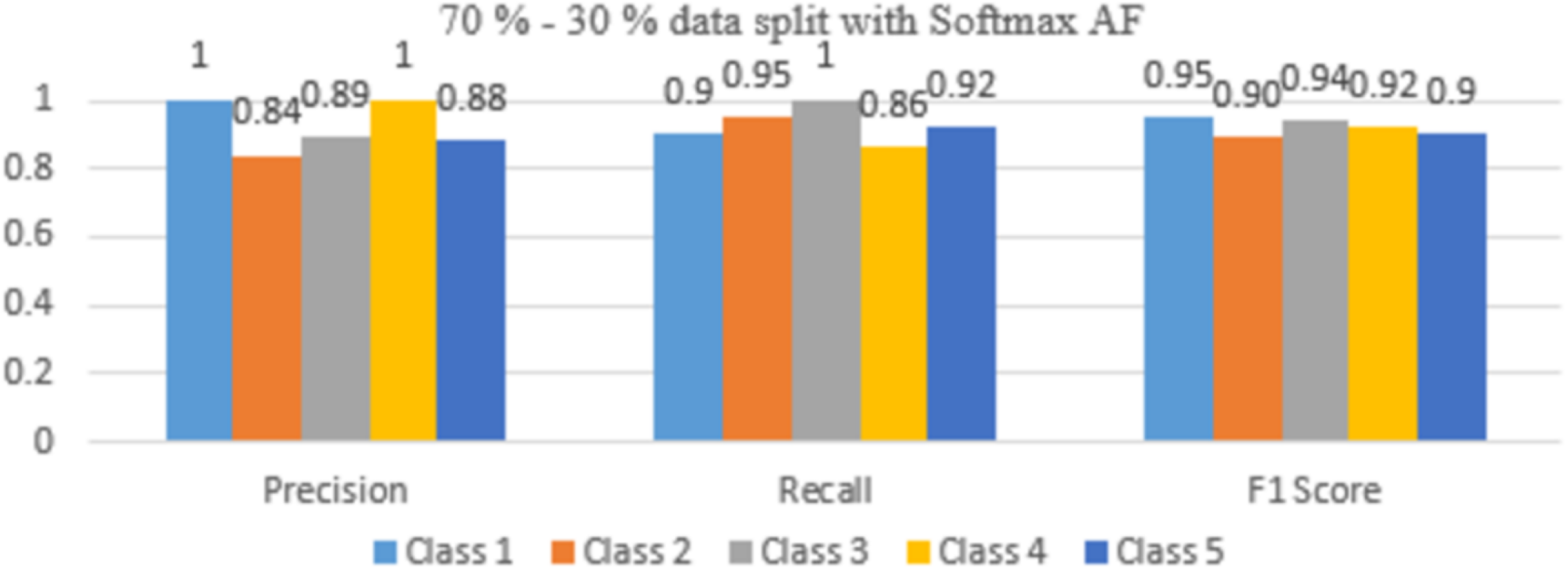
Classification Evaluation Metrics for 70–30% data split with softmax activation function.

The results are shown from Figs. 7 to 10 represent the values of precision, recall, and F1 score for each rice disease class when used with 70–30% training and testing split of dataset parameters. The results are shown for sigmoid, tanH, ReLU, and softmax functions respectively.

In Fig. 7, Class 5 rice diseases False Smut are more accurately identified as compared to other classes of diseases followed by Class 1 of healthy conditions. The rest classes are moderately classifying the diseases.

In Fig. 8, Class 5 and Class 3 are identified accurately with 0.83 and 0.79 F1 scores respectively. These values are low as compared to sigmoid AF. Classes 1,2 and 4 are classified with F1 score accuracy between 0.73 to 0.76 values.

Figure 9 represents that with ReLU AF, only classes 3 and 4 have F1 scores above or equal to 0.90 value. Class 1, 2 and 5 have scores within the range of 0.86 to 0.89 so it needs to be improved.

By observing Fig. 10, the F1 score metrics for all the classes are above 0.90 which means softmax AF when combined with 70–30% dataset split gives maximum accuracy.

### Comparison of activation functions with 80–20% dataset split

**Figure 11:**
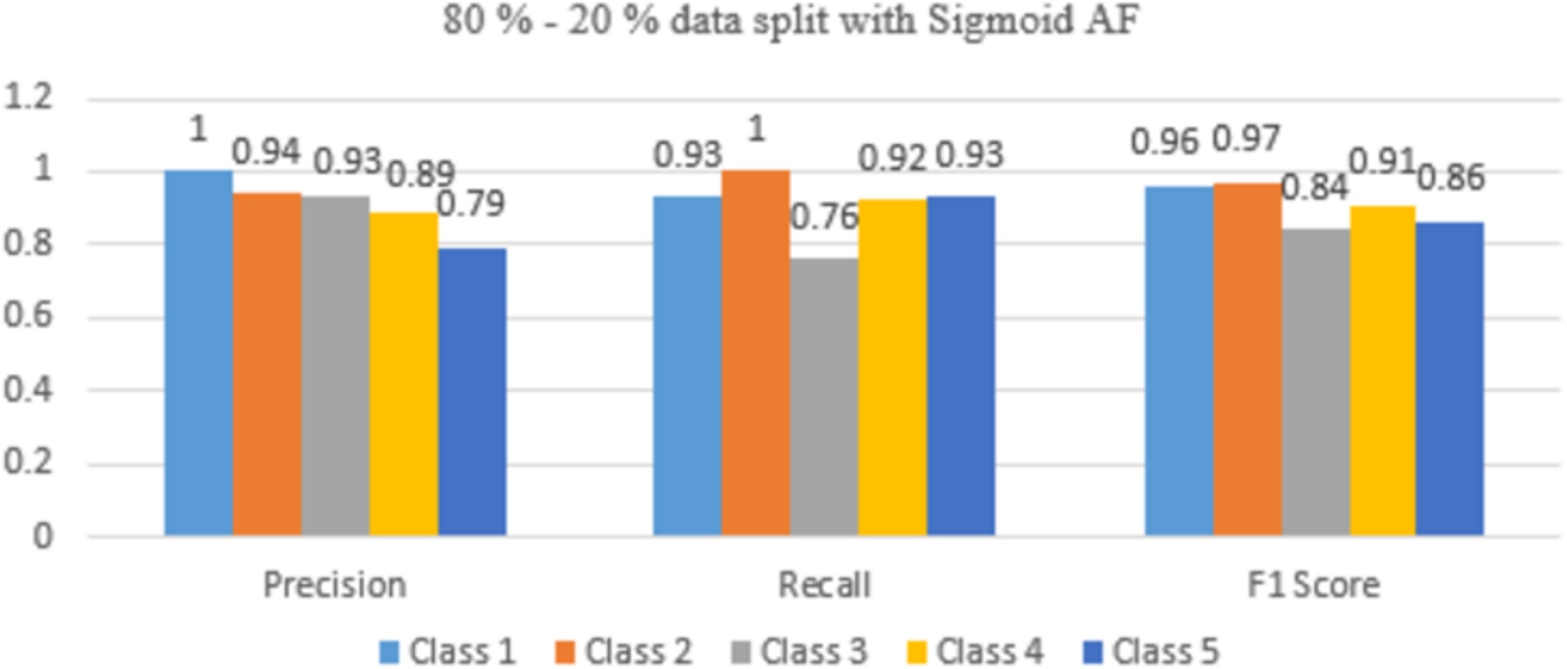
Classification evaluation metrics for 80–20% data split with the sigmoid activation function.

**Figure 12:**
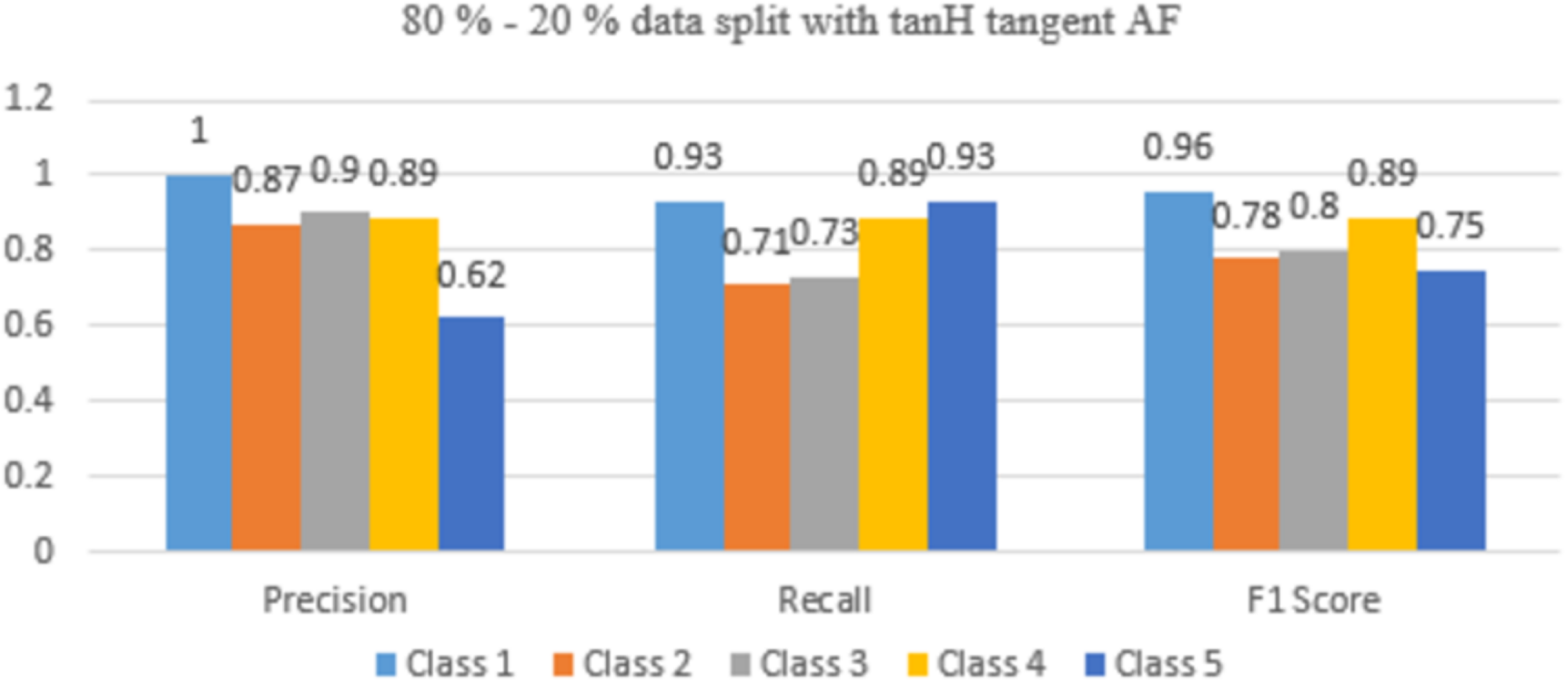
Classification evaluation metrics for 80–20% data split with the tanH activation function.

**Figure 13:**
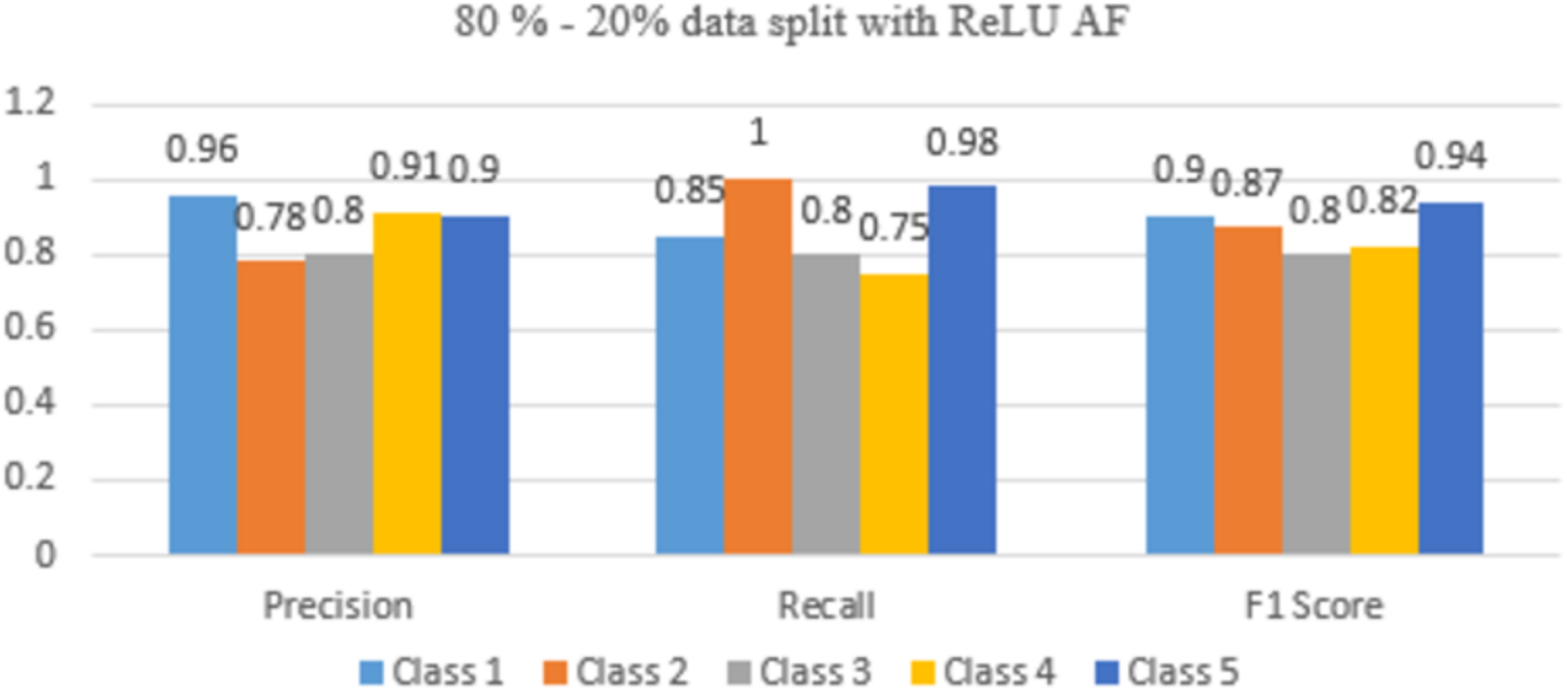
Classification evaluation metrics for 80–20% data split with the ReLU activation function.

**Figure 14:**
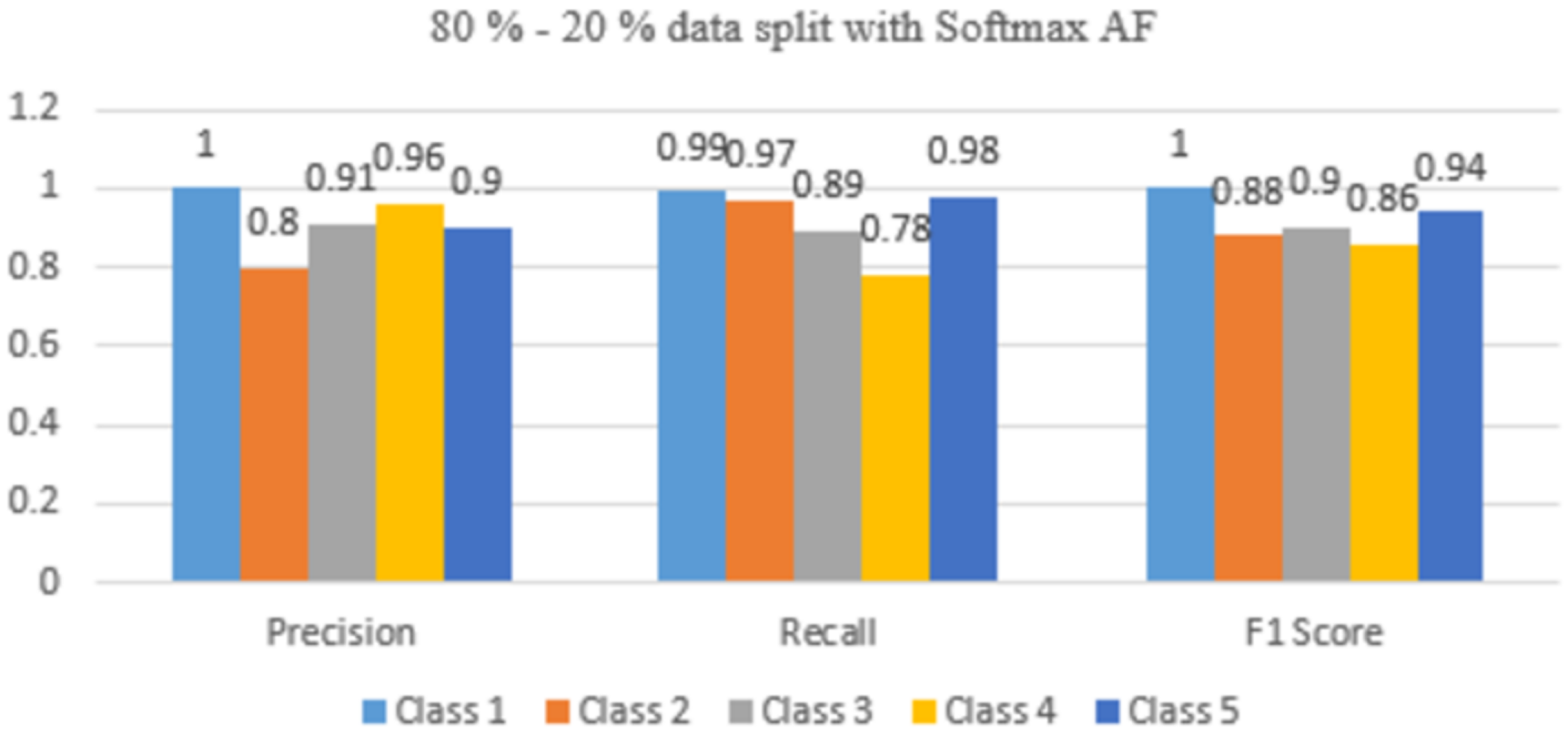
Classification evaluation metrics for 80–20% data split with the softmax activation function.

The results are shown from Figs. 11 to 14 represent the values of Precision, Recall, and F1 score for each rice disease class when used with 80–20% training and testing split of dataset parameters. The results are shown for sigmoid, tanH, ReLU, and softmax functions respectively.

Figure 11 represents that Class 1, 2, and 4 are classified with maximum accuracy but Class 3 and 5 are moderately classified. If compared to a 70–30% split, performance is better.

In Fig. 12, Class 5 is misclassified, reciprocally with 70–30% split Class 5 was more accurately classified with tanH AF.

In Fig. 13, Class 1, and 5 have good accuracy with ReLU AF as compared to Class 2, 3 and, class 4.

Figure 14 depicts that Class 1,3 and 5 are achieving good accuracy however Class 2 and 4 are still misclassified.

After comparing both the cases, the solution that is obtained must be optimal which means the model should be able to predict the occurrence of all diseases with maximum accuracy. Softmax AF gives maximum accurate results for predicting all five classes of rice diseases when data was split with 70–30% capability. It can be concluded that the proposed model performs best when the model was applied to 70–30% training and testing sets. The model performance decreased when it was applied to 80–20% data split.

**Figure 15:**
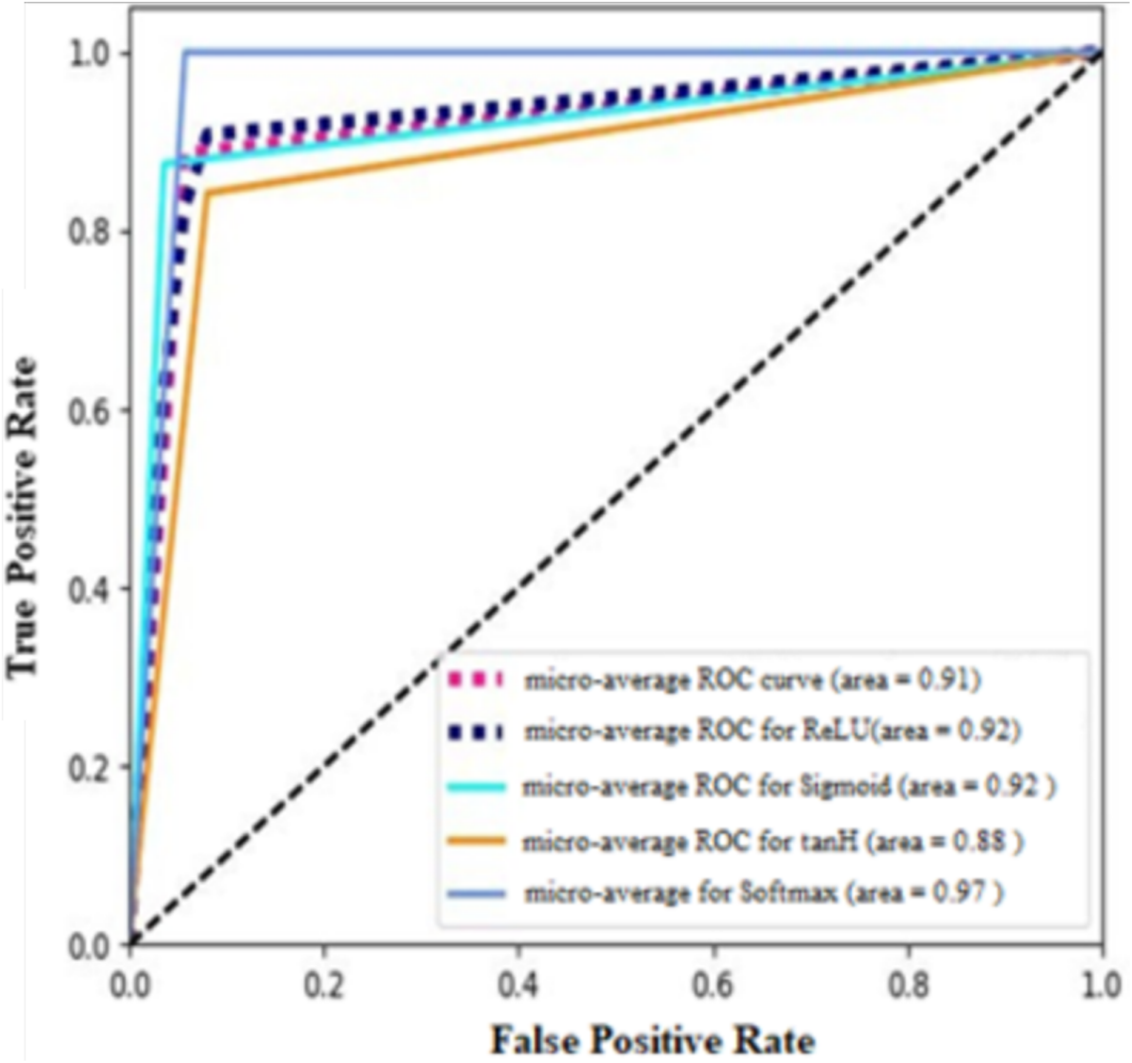
Micro-average ROC curve for the activation functions used with two cases of data split.

Receiver operating characteristics (ROC) for four different types of AF implemented in this research paper are shown in Fig. 15. It calculates the micro-average curve for all the activation functions. It clearly shows that softmax AF produces maximum accuracy as the ROC curve is highly inclined towards true positive rate. Also, tanH AF misclassifies the instance in most cases among the four classifiers demonstrated in the paper as its curve is inclined more towards false positive rate.

Table 4 shows the comparison of the overall accuracy of four activation functions with two different cases of dataset division.

**Table 4:** Comparison based on overall accuracy of the model for different activation functions.

Dataset split	Sigmoid	Hyperbolic tangent	ReLU	Softmax
70–30%	79.77%	77.15%	89.31%	92.15%
80–20%	90.13%	82.85%	86.47%	90.98%

It can be concluded that by making use of softmax AF the model achieves a maximum prediction overall accuracy of 92.15% which is the highest of all. The model performs best at data split 70–30% of training and testing sets as compared to model when applied to 80–20% data split. ReLU activation function performs in an average manner for both the cases of data split. It performs better when the training set is larger. The sigmoid activation function gives better performance with 80–20% data split. Tanh activation function has demonstrated poor performance when compared with all other activation functions implemented in the research paper. Softmax activation function outperforms when applied with 70–30% data split.

## Discussion

A common technique to identify and predict crop diseases is to use image processing techniques where images of plant leaves are given as input to the model. Image processing is used to measure the disease-affected area by calculating the deviation in the color of the affected area. Image classification is responsible for differentiating between an infected plant leaf and a healthy plant leaf. Identification of whether the plant is affected or healthy is made. The severity of the disease is also rated. However, using image processing techniques has its limitation of unable to predict the diseases well beforehand. Another method to predict crop diseases by correlating weather information. In our analysis, we have used ANN which is an artificial intelligence model with different activation functions which resulted in high precision and recall classification.

The dataset was divided into two cases and the model was implemented for all four activation functions for both the data division cases. The model gives good overall accuracy when used with 70–30% data split as compared to 80–20% split.

In our model, regression and classification techniques are combined to attain the highest accuracy. For this, the optimized number of the epoch was identified. The model was executed for different epochs ranging from 100 to 900. When 100 to 400 epochs were considered it gave more MAE while if the epoch value is between 600 to 900 it gave less MAE but it required more training time. Therefore, using 500 in epochs becomes an optimal solution as it finds a balance of less MAE and minimum training time. The optimized batch size is 32 as reducing the batch size obtained linear line to predict the data while increasing the batch size resulted in the problem of resource exhaustion. ReLU AF performs well in the regression model where agro-meteorological normal are predicted. In the classification model, softmax AF gives maximum accuracy as compared to sigmoid, tanH, and ReLU. softmax AF is best when multiple class classification is to be done.

We used MAE as regression evaluation parameter and Precision, Recall, F1-score, and Accuracy as a classification evaluation parameter. For our model, precision and recall are equally essential. So instead of individually focusing to achieve good values for each parameter, it is better to achieve the balance between precision and recall, so we have aimed for a high F1-score and that would imply strong precision and a strong recall value as well. With softmax, we have achieved a high F1 score for all classes of rice diseases. ROC curve of softmax AF is inclined towards TPR while tanH AF is more inclined towards FPR which means it misclassifies the instances more as compared to other AF.

The results represent that the performance of softmax AF is better than sigmoid, tanH, ReLU for all five rice disease classes considered in the research paper. The softmax AF have reduced the number of false positive and false negative prediction and achieves the highest accuracy in multiclass classification.

There is a huge plethora of AI algorithms available to solve realistic problems. The most commonly used ANN techniques are multi-layer perceptron, radial basis function, and support vector machines (Sumathi et al., 2021). Each algorithm has its pros and cons. It is necessary to choose a type of network based on the nature of the problem. Still, we have tried to compare different models present in the literature. This is depicted in Table 5.

**Table 5:** Comparison of the proposed model in the manuscript with the existing model.

Authors	Crop/fruit/plant/grain/vegetables	Diseases	Input	AI approach	Performance evaluation metrics	Output of the model
Badnakhe et al. (2018)	Citrus	Gummosis	Temperature, Humidity, Rainfall, Soil Moisture, Soil Temperature, Leaf Area Index Chlorophyll	Support vector regression (SVR), Multilinear regression (MLR)	RMSE (SVR) =0.9061 RMSE (MLR) =0.8518	Presence of Gummosis disease
Orero & Toroitich (2017)	Potato	Late Blight	Temperature, humidity values from sensors	BPNN	Accuracy = 94%	3 classes of disease severity: high, medium and low
Pérez-Ariza, Nicholson & Flores (2012)	Coffee	Cercospora, Rust	monthly incidence of the rust, a daily temperature summary, humidity, wind speed, solar radiation	BN, Naive Bayes, DT	Confusion Matrix, Error Rates	Disease incidence
Cabrera Ardila, Alberto Ramirez & Prieto Ortiz (2020)	Mango	Anthracnose	Spectral analysis	Random Forest (RF), SVM with Principal Component Analysis (PCA) and Linear Discriminant Analysis(LDA)	Accuracy with LDA = 91%	Healthy, diseased or asymptomatic.
Ahmadi et al. (2017)	Oil palm	Ganoderma	Spectral analysis	ANN	Accuracy=83%	Severity of disease
Nettleton et al. (2019)	Rice	Blast	Temperature, humidity, leaf wetness	M5Rules, LSTM	R=0.70, R^2^ =0.50, MAE=0.75	Severity index of Disease
Ramakrishnan & Sahaya (2015)	Groundnut	Cercospora stages	Images of groundnut leaf	BPNN	Accuracy = 97.41	Diseases Severity
Zhang & Meng (2011)	Citrus	Canker, black spot, scab, and melanose	Images of citrus leaf	Ada Boost, RBN, k-NN, SVM	Accuracy = 88%, 73.25%, 69.25%, 63%	Identification of diseases
Bandi (2013)		Greasy spot, Scab, Melanose and Healthy	Images of leaf affected with each category of citrus disease and healthy leaf	k-NN, NB, LDA, RFT	Accuracy = 77.5%, 95%, 98.75%, 97.5%	Classification of diseases
Ghaffari et al. (2010)	Tomato	Powdery mildew, spider mite	Volatile Organic Compounds (VOCs)	MLP,RBF, Learning Vector Quantization (LVQ)	Accuracy RBF = 94% MLP = 96% LVQ = 98%	Classification of healthy and infected tomato plants
Abdulridha et al. (2019)	Avocado	Laurel Wilt, Phytophthora Root Rot, Iron and Nitrogen Nutrient Deficiencies	Images of avocado trees	KNN, MLP	Classification Accuracy Symptomatic stage = 83–93% Asymptomatic stage = 79–85%	Asymptomatic stage and symptomatic stage.

After comparing the above literature with the proposed literature, it can be concluded that most of the researchers have focused on plant disease detection, classification, and identifying disease severity levels. More attention is required on plant disease prediction which has been addressed in the proposed model. A range of work exists in exploring diseases occurring on fruits while diseases occurring on food grains are less researched. In the proposed model food grain rice is considered. The images of leaves are used to identify plant diseases however the proposed model uses the agro-meteorological parameters for prediction of diseases. Most of the models in the literature have been proposed for foreign climatic conditions while the proposed model focuses on the Indian climatic conditions. The results from the existing methods are accurate but not up to the extent that they can be considered for real life agriculture diseases.

In the lower-middle-income countries like India, the farmers are still lagging when it comes to digitization and automation in the agricultural domain. The proposed systems can help farmers to predict the occurrence of the disease accurately well in advance and take corrective and preventive measures. This will reduce huge economic losses caused due to diseases in crops. The model works accurately to predict limited fungal and bacterial diseases on rice crops for certain geographical locations. This can be considered as one of the limitations of the proposed model. The model proposed in this research could be more practical if diseases on crops other than rice can be predicted well in advance for different geographical locations. Further to this, deep learning techniques such as LSTM, RNN can be used to build the proposed model which may further enhance performance evaluation metrics of the proposed model. In future work, the image dataset of the rice leaf can be used for rice disease prediction, identification, and classification which is not present in the proposed model. Moreover, an advisory bulletin can be employed in the model that will be helpful to the farmers.

## Conclusions

The model developed is a combination of regression and classification. The regression model predicts the values of agro-meteorological parameters with a mean absolute error of 0.46 which is minimum. ReLU activation function gives better mean absolute error as compared with others. It can be concluded that ReLU activation functions are ideal for predicting climatic parameter values. For the classification model, the accuracy achieved is 92.15% which is the highest amongst all. Thus, it can be concluded that the proposed model helps to identify the healthy condition and four different types of rice diseases namely rice blast, bacterial blight, smut, and Brown spot well in advance based on agro-meteorological parameters so that economic loss can be reduced. The softmax activation function gives better accuracy in classifying rice diseases. Precision, recall, F1 score, and accuracy are the evaluation parameters used for classifying diseases. Thus artificial neural network approach is best amongst the plethora of algorithms available in artificial intelligence. Further, it can be applied to different types of crops, for different geographical locations to predict different types of diseases well in advance.

## Supplemental Information

10.7717/peerj-cs.687/supp-1 Supplemental Information 1 Prediction of the agro-meteorological parameters using Regression. Each data point is the average of the agro-meteorological parameters values which is collected week wise as per Indian Meteorological Department and Regressor is applied to predict values.

10.7717/peerj-cs.687/supp-2 Supplemental Information 2 Python code for Regression to predict values. ANN model is built where, model is trained and tested with 70–30% data split with Activation functions.

## Additional Information and Declarations

### Data Availability

The following information was supplied regarding data availability: The data and code are available in the Supplemental Files.
